# First Cases of *Candida auris* in a Referral Intensive Care Unit in Piedmont Region, Italy

**DOI:** 10.3390/microorganisms10081521

**Published:** 2022-07-27

**Authors:** Silvia Corcione, Giorgia Montrucchio, Nour Shbaklo, Ilaria De Benedetto, Gabriele Sales, Martina Cedrone, Davide Vita, Cristina Costa, Susanna Zozzoli, Teresa Zaccaria, Carlo Silvestre, Rossana Cavallo, Luca Brazzi, Francesco Giuseppe De Rosa

**Affiliations:** 1 Department of Medical Sciences, Infectious Diseases, University of Turin, 10126 Turin, Italy; nour.shbaklo@edu.unito.it (N.S.); ilaria.debenedetto@unito.it (I.D.B.); davide.vita@unito.it (D.V.); francescogiuseppe.derosa@unito.it (F.G.D.R.); 2 School of Medicine, Tufts University, Boston, MA 02111, USA; 3 Department of Surgical Sciences, University of Turin, 10126 Turin, Italy; giorgia.montrucchio@unito.it (G.M.); gabriele.sales@unito.it (G.S.); martina.cedrone@unito.it (M.C.); luca.brazzi@unito.it (L.B.); 4 Department of Anaesthesia, Critical Care and Emergency, Città Della Salute e Della Scienza Hospital, Corso Dogliotti 14, 10126 Turin, Italy; 5 Microbiology and Virology Unit, University Hospital Città Della Salute e Della Scienza di Torino, 10126 Turin, Italy; cristina.costa@unito.it (C.C.); tzaccaria@cittadellasalute.to.it (T.Z.); rossana.cavallo@unito.it (R.C.); 6 Direzione Sanitaria, Presidio Ospedaliero Molinette, AOU Città della Salute e della Scienza di Torino, 10126 Turin, Italy; szozzoli@cittadellasalute.to.it; 7 Health Management Molinette Hospital, University Hospital Città della Salute e della Scienza di Torino, 10126 Turin, Italy; csilvestre@cittadellasalute.to.it

**Keywords:** *C. auris*, infection control, ICU, critically ill, antifungal stewardship, COVID-19

## Abstract

*Candida auris* is an emerging healthcare-associated infection that can easily cause dissemination in hospitals through colonizing the skin and contaminating environmental surfaces, especially in Intensive Care Units (ICU). Difficulties with identification of this organism, uncertainty about routes of transmission and antifungals resistance have impacted significantly outbreak detection and management. Here, we describe our experience with colonization/infection of *C. auris* among critically ill patients, admitted to a referral ICU of a University Hospital, in a transitional period (July 2021–March 2022) between management of non-COVID-19 and COVID-19 patients due to the reconversion of the ICU between two waves. A total of 8 patients presented colonization from *C. auris*, and two of them developed invasive infection from *C. auris*. The fungal pathogen was cultured from different sites: the skin (7 isolates), urine (2), respiratory tract (1), blood (1). The median time from admission to first detection is 24 days with 100% of patients requiring mechanical ventilation. All 8 patients received broad-spectrum antibiotic therapy for bacterial infections before identification of *C. auris*; 62.5% of the patients had prior antifungal exposure; 87.5% received steroids; 37.5% patients used immunomodulatory; and 75% had severe COVID-19 illness prior to *C. auris* identification. Only two cases (25%) were treated with antifungals as *C. auris* infections (1 patient for suspected UTI; 1 patient with candidemia). Infection control measures, including rapid microbiological identification, contact isolation, screening of contacts, antisepsis of colonized patients, dedicated equipment, cleaning and disinfection of the environment and subsequent follow-up sampling, remain essential in critically ill patients. Our experience highlights the importance of establishing a multidisciplinary model and bundling of practices for preventing *C. auris*’ spread.

## 1. Introduction

Since its first description in 2009, *Candida auris* has been a serious public health threat. Invasive Fungal Infection (IFI) caused by this species has been described in more than 40 countries [1]. Due to its high multidrug resistance [1], transmissibility and long persistence in the hospital environments [2,3,4], it is considered a serious global threat causing outbreaks and deep-seated infections [5].

*C. auris* combines all the essential characteristics for a pathogen to pose a threat to public health: potential to spread through horizontal transmission; ability to cause serious and life-threatening infections; multi-resistance profile and limitations for optimal treatment [1].

Nowadays, little evidence on its pathogenicity and the complex host–pathogen interactions is available [1]. Progress in its identification with definite diagnostic molecular or spectrometry tools is crucial but is not equally available in hospitals and countries.

Several risk factors were related to the development of infection, especially in previous colonized patients, and treatment options remain really scant [1]. In 2019, the Center for Disease Control and Prevention of the United States (CDC) considered *C. auris* infection an urgent threat for international public health in the field of multidrug resistant microorganisms [6]. New epidemiological alerts have been released in view of the increase in healthcare-associated *C. auris* cases in the context of the COVID-19 pandemic, worldwide and in Italy [7,8,9,10].

The latest ECDC survey reports cases of *C. auris* in 9 countries in the EU/EEA, including Italy, where an outbreak in Liguria (North-West Italy) is still ongoing [11].

In a worldwide retrospective study of clinical characteristics of *C. auris*, there was a higher proportion of men, premature babies and elderly people [12]. The proportions of patients with underlying diseases such as diabetes, kidney disease, trauma and ear disease were also high. More than half of patients had a history of central venous catheter use and a history of broad-spectrum antibiotic use. As previously said, recently, a sharp rise in new cases of *C. auris* colonization and infection has been reported, especially during the ongoing COVID-19 pandemic probably due to the increased vulnerability of SARS-CoV-2-infected patients with severe respiratory and immune damage [13].

Several diagnostic and therapeutic challenges have been reported with *C. auris*: first, *C. auris* may be misidentified by conventional phenotypic methods; second, most of th isolates are resistant to fluconazole, a subset of the *C*. *auris* strain that has high minimum inhibitory concentrations (MICs) to amphotericin B and echinocandins, and some *C*. *auris* isolates are resistant to all antifungal classes [14]. In terms of treatment, it constitutes the only fungal species able to be resistant to azoles, amphotericin B and echinocandins, although clinical data seem to suggest a more differentiated pattern of antifungal susceptibility [5,14].

From an infection-control perspective, daily cleaning and disinfection are recommended for patients’ rooms because *C. auris* persists on surfaces. The Center for Disease Control and Prevention (CDC) recommends using an Environmental Protection Agency (EPA)-registered hospital-grade disinfectant that is active against *C. auris* listed on List P [15]. Moreover, screening contacts of identified cases for *C. auris* is essential to contain the organism’s spreading.

We report here the first cases of *C. auris* in our ICU, among critically ill patients, in a transitional period between management of non-COVID-19 and COVID-19 patients, focusing on clinical characteristics of eight ICU cases, microbiological detection methods, infection control procedures and mortality rates.

## 2. Materials and Methods

We describe eight cases of colonization or infection caused by *C. auris* observed between July 2021 and March 2022 in an 8-bed intensive care unit of a 1200-bed academic hospital with primary and secondary referral (AOU Città della Salute e della Scienza, Turin, Italy).

Surveillance cultures (urine culture, tracheal aspirate, rectal swab) are performed weekly. *C. auris* was not routinely sought, except for patients with previous contiguity with infected/colonized cases. In those cases, surveillance cultures (urine culture, tracheal aspirate, rectal swab) were performed weekly. The study was approved by the Local Ethical Committee (Prot.n. 0008191).

### 2.1. Environmental Sampling

SRK Copan swabs (Brescia, Italy) were used for environmental sampling. Samples from healthcare workers’ hands were collected after contacting an infected or colonized patient. Twenty swabs from the hands, one from a cell phone and three from hands with gloves were collected.

Surface swabs were collected to evaluate the efficacy of cleaning interventions. Forty-two samples were obtained from high-touch surfaces such as doorknobs, light switches, keyboards, screens, difficult-to-disinfect sites such as and machine-equipped zones. To avoid false results related to health-care workers, samples were collected during 3 different working days.

The swab was rotated between the thumb and forefinger during the sweeping action to maximize the uptake of the surface material. The samples were transported to the laboratory for analysis within 2 h in a cool box at 1–4 °C. The samples had the possibility to be refrigerated at 2–8 °C for up to 24 h before laboratory analysis. In the laboratory, the swab was mixed using the vortex to release sample material and make an even suspension before the culture.

### 2.2. Microbiological Detection

Culture-based approaches remain the mainstay of the laboratory diagnosis of *C. auris. Candida* isolates from clinical swabs were plated on BD Sabouraud Agar with gentamicin and chloramphenicol agar plates (Becton Dickinson GmbH, Heidelberg, Germany) and identified using chromogenic agar with five days at 37 °C implemented for the incubation protocol (Brilliance Candida Agar, Thermo Scientific, Basingstoke, UK). Non-*C.albicans* isolates including *C. auris* were identified to the species level by MALDI-TOF (Bruker, Bremen, Germany) using the Biotyper v4.1.100 software. MIC determination was conducted by microbroth dilution according to the standard EUCAST with the commercial method MICRONAUT-AM antifungal agents MIC (Bruker, Bremen, Germany).

### 2.3. Infection Prevention and Control Interventions

Following the finding of a positive urine culture for *C. auris* of a patient during surveillance standard screening, the Infection Control Office proceeded to identify an operative protocol capable of defining other potential cases and recommended a rigorous application of control measures. These measures included: rapid microbiological identification, isolation or cohort of cases, screening of contacts, antisepsis of colonized patients, cleaning and disinfection of the environment and subsequent follow-up sampling, according to available guidelines [16,17,18].

To remind about contact isolation, an educative poster was put with an indication to reduce the number of entries to the minimum, even to healthcare personnel. Educational activities were conducted about monitoring, contact precautions for non-ICU staff, while it was also recommended to use dedicated, disposable equipment where applicable and immediately disinfect/sterilize reusable equipment. Skin antisepsis was conducted for colonized patients with disposable wipes of non-alcoholic 2% chlorhexidine gluconate solution on alternate days.

Screening of patients was performed from axillary, inguinal and tracheal and nostril swabs. Screening was conducted for all close contacts including: all patients who shared the same hospital room in the same hospitalization period with the index case; those who were cared for by the same healthcare staff who handled the index case; and those who occupied the same bed of the index case despite cleaning and disinfection. Screening was repeated weekly in contacts who had a negative result. In addition, all patients hospitalized in the same period were weekly screened for fungi in respiratory and urine samples. In case of the positivity of fungal growth, a specific culture was carried out to rule out *C. auris*.

## 3. Results

### 3.1. Patient Characteristics

Overall, eight patients with *C. auris* colonization or infection were observed. Four (50%) patients were male; the median age of the population was 57.5 years old (IQR 52–61), with several comorbidities. Population characteristics are described in Table 1. Of note, 75% of patients were admitted to the ICU due to COVID-19-related acute respiratory distress syndrome (ARDS). Considering patients’ severity, the median Charlson Comorbidity Index was 3.5, while the median SOFA score was 7 (IQR 6–9), and the median SAPS II at ICU admission was 36.5 (IQR 30–40). The median hospital of stay was 41 days (IQR 26.5–83), and the median ICU stay was 33 days (IQR 24.5–50). The median sites of *C. auris* colonization were 1 (IQR 1–3), and the median time from admission to first *C. auris* detection was 24 days, with 100% of patients requiring mechanical ventilation. In 5 patients (62.5%), skin was the site of *C. auris* isolation; in 2 (25%), it was urine; in 1 (12.5%), it was the respiratory tract. The isolates were resistant to fluconazole with a MIC >128 μg/mL. The MIC was interpreted according to the criteria of the European Committee on Antimicrobial Susceptibility Testing (EUCAST) of non-species-related breakpoints for *Candida*.

All eight patients received broad-spectrum antibiotic therapy for bacterial infections before identification of *C. auris*; 62.5% (5) had prior antifungal exposure (with 4/5 of them having previous colonization of other Candida species); 87.5% received steroids; 37.5% of patients used immunomodulatory drugs.

Out of eight patients with colonization, two cases (25%) were simultaneously infected and treated with antifungals as a part of empirical broad spectrum antibiotic treatment. In fact, one patient had candidemia due to *C. auris*, and another one presented with persistent fever and a possible urinary tract infection. Overall, the crude 28-day mortality rate was 50%.

### 3.2. Surveillance

In total, 66 environmental samples were collected. A total of 44 were positive for Gram-positive polymicrobial flora. However, they were normal cutaneous microbiota with a non-significant microbial load. Four positive samples of *Enterobacteriaceae* were revealed from closets in the corridor, which is considered as a clean area, the trolley and drawers. Of them, three had a bacterial load >50 CFU and were identified as *Klebsiella pneumonia carbapenemase* (KPC). Swabs from health-care workers’ hands were negative. Positivity for *C. auris* was never found on the surfaces investigated.

### 3.3. Infection Control

**Figure 1:**
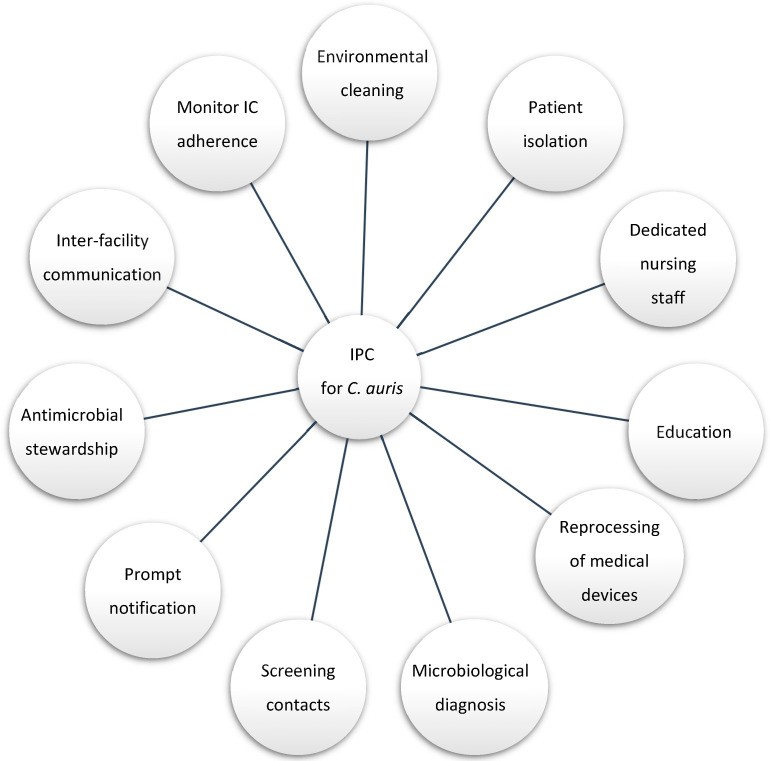
Infection control practice (IPC) and stewardship cornerstone for *C. auris* management.

Daily environmental cleaning and disinfection were intensified for up to four times a day and in case of spills or visible dirt. High-touchable surfaces were disinfected using a chlorine-based solution with concentrations not lower than 1000 ppm. Terminal cleaning at the patient’s discharge was conducted with a dilution of 5000 ppm, followed by a nebulization with hydrogen peroxide [15,16,17]. Materials and furnishing that are metallic and/or intolerant to chlorine were treated with a disinfectant based on didecyldimethylammonium chloride and chlorhexidine digluconate. Finally, they were disinfected by passing a cloth soaked in chlorine-based solution. Follow-up sampling was carried post-disinfection. Figure 1 summarizes the multi-disciplinary infection control measures conducted to contain the spread of *C. auris*.

## 4. Discussion

The case series of *C. auris* here reported in the ICU represented to date the first outlined phenomenon in Piedmont, Italy.

Considering our cohort, it is crucial to mention the severity of the patients, such as the prolonged hospital stay of colonized/infected patients. Even though no risk factor was identified with this analysis, two-thirds of patients had COVID-19-related ARDS, requiring mechanical ventilation in line with what was recently reported by Briano et al. [13]. This finding suggests that a therapy with steroids or immunomodulatory agents could be a risk factor for *C. auris* isolation. Moreover, the use of broad-spectrum prior to *C. auris* isolation in all cases might suggest it as risk factor for *C. auris* acquisition similar to documented risk factors for *Candida* spp. invasive infections [19,20,21].

We also underline that, in our case series, many patients were immunosuppressed for prolonged hospitalizations and multiple complications, and presented simultaneous or previous presence of other infections, especially from Gram-negative, difficult-to-treat pathogens. In this sense, in addition to the already mentioned use of antimicrobials, and relevance of infection control measures, other factors may have influenced the selection of the microbiome, such as parenteral/enteral nutrition or previous bacterial colonizations or infections. Actually, the fact of colonization with *C. auris* is linked to the frailty of the patients due to comorbidities, invasive procedures and long hospital stays.

Nonetheless, it is not elucidated if a prior use of antifungals agents including echinocandins or a previous *Candida* spp. infection might induce a *C. auris* colonization or if it is rather due to clonal dispersion phenomenon as recently proven by a study on *C. parapsilosis* isolates harboring the Y132F ERG11 gene substitution that demonstrated that resistance to fluconazole was not attributable to prior azole use but rather to a group of fluconazole-resistant *C. parapsilosis* that have become endemic [22]. In our case series, two-thirds of *C. auris* patients received previous antifungals, of which more than a half were echinocandins. Interestingly, even though two isolated strains of *C. auris* showed in vitro resistance to all antifungals including echinocandins, only one case had a prior exposure to echinocandins. Although the development of infection and candidemia has been associated with multisite colonization as an independent risk factor in critically ill patients [13], in our cases, we did not observe a higher frequency of colonized sites in the two patients who developed infections.

One possible explanation for *C. auris*’ spread at our institution could be the high rate of transferred patients from peripheral hospitals during the COVID-19 pandemic as well as the high number of incoming patients from other peripheral regions for transplant evaluation and procedures, in which *C. auris* was previously described [13]. An essential aspect in infection control might be in fact the management of patient transfers between different hospitals: In case of the need for inter-hospital transfers, it is important that all hospitals can guarantee an adequate diagnostic standard.

Limitations of our report include the lack of comparison with controls not colonized by *C. auris* to confirm if colonization is a robust risk factor. In addition, the limitation due to environmental sampling meant that it was not possible to evaluate all possible surfaces that may have contributed to the spread. It is probable that the dissemination of the case series is an outbreak due to different factors such as: organism resistance, hospital transfers, invasive procedures and critically-ill patients, invasive procedures and breaches in infection control. Yet another limitation is that we could not identify the source of the transmission among different patients and molecularly or genomically type the similarity of the clone to confirm the modality of the spread.

## 5. Conclusions

In conclusion, we report here the first case series of *C. auris* in a regional referral ICU. In our case series, patients developed *C. auris* colonization after a long hospital stay in patients transferred from different hospitals with several comorbidities and previous bacterial infections. Few invasive infections were reported, supporting the finding that *C. auris* is a relevant infection control issue especially in the setting of fragile, critically ill patients, but its clinical role in determining invasive infections needs further data. Mortality in colonized patients was high (50%); however, the low number of invasive infections does not support a correlation between the pathogen and mortality.

## Figures and Tables

**Table 1:** Characteristics of *C. auris* colonized/infected patients.

ID	Sex, Age	Hospital Stay (days)	ICU stay (days)	Death	Comorbidities	COVID-19	Site of Isolation (1)	Site of Isolation (2)	Subsequent Infection Type	Antifungal Treatment for *C. auris*	Mechanical Ventilation	Steroids	Immuno-Modulatory Agents	Previous Broad-Spectrum ATB	Previous Antifungal tp	Other Infections	Microorganism
1	M 44	47	35	Yes	Autoimune disease, respiratory disease, smoker	No	skin	skin	Colonization	No	Yes	Yes	Yes	Yes	Yes	CAP	*S. marcescensPJP*
2	F 58	35	31	No	Smoker, HTA	No	urine	skin	Colonization	No	Yes	No	No	Yes	No	VAP	*P. aeruginosa, S. marcescens*
3	M 64	100+	35	No	N/A	Yes	skin	-	Colonization-Suspected UTI	Yes—Anidulafungin	Yes	Yes	No	Yes	No	VAP/BSI	*M. morgani/KPC, E.faecalis*
4	M 64	16	14	Yes	Respiratory disease, smoker, HTA, DMNID	Yes	skin	-	Colonization	No	Yes	Yes	No	Yes	Yes	VAP/BSI/CAPA	*A.baumannii + KP/E.faecium* VRE
5	F 49	25	22	Yes	Respiratory disease, HTA, DMNID, autoimmune disease	Yes	skin	-	Colonization	No	Yes	Yes	No	Yes	No	VAP	*A.baumannii* + KP ESBL
6	M 57	28	27	Yes	Autoimmune disease	Yes	urine	-	Colonization	No	Yes	Yes	Yes	Yes	Yes	VAP/BSI	*P. aeruginosa/* *C. albicans*
7	F 55	100+	100+	No	HTA, hemathological disease, malignancy	Yes	respiratory tract	blood	Colonization-Infection	Yes—Anidulafungin, Ambisome	Yes	Yes	Yes	Yes	Yes	VAP/BSI	*KPC/C. albicans*
8	F 58	66	65	No	respiratory disease, HTA, DMNID, autoimmune disease	Yes	Skin	-	Colonization	No	Yes	Yes	No	Yes	Yes	VAP/BSI	MRSA/KPC

ICU, Intensive Care Unit; ATB, Antibiotics; TP, Therapy; HTA, Arterial Hypertension; DMNID, Diabetes Mellitus Not Insulin Dependent; CAP, Community Acquired Pneumonia; VAP, Ventilator Associated Pneumonia; UTI, Urinary Tract Infection; BSI, Bloodstream Infections; CAPA, COVID-19 Associated Pulmonary Aspergillosis; PJP, Pneumocystis Jiroveci Pneumonia. KPC, Klebsiella Pneumoniae KPC; KP, *Klebsiella pneumonia*; MRSA, Methicillin Resistant *Staphylococcus aureus*; ESBL, Extended Spectrum Beta-Lactamase; VRE, Vancomycin Resistant *Enterococcus*.

## Data Availability

Due to patient confidentiality, raw data will be available upon request with a compelling reason.
